# 164. BioFire FilmArray Pneumonia Panel Implementation: Examining Appropriate Usage and Opportunities for Diagnostic Stewardship

**DOI:** 10.1093/ofid/ofac492.242

**Published:** 2022-12-15

**Authors:** Casey S Zelus, Trevor C Van Schooneveld

**Affiliations:** University of Nebraska Medical Center, OMAHA, Nebraska; University of Nebraska Medical Center, OMAHA, Nebraska

## Abstract

**Background:**

The BioFire FilmArray Pneumonia Panel (PnaP) is a novel diagnostic tool for rapid diagnosis of lower respiratory tract infections. However, appropriate usage and optimal implementation strategy in the clinical setting remain poorly characterized. The PnaP assay was introduced along with institutional guidance restricting use to intensive care units (ICUs) and Infectious Disease and Pulmonary consultative services. Examining guideline compliance and clinically appropriate PnaP usage highlights the role of diagnostic stewardship in improving management of pneumonia in hospitalized patients.

**Methods:**

PnaP testing was obtained retrospectively from hospitalized adults over a 6-month period (January 1 - June 30, 2021). Outpatient and pediatric (< 18 years of age) testing was excluded. Appropriate use criteria based on institutional guidance are defined in Figure 1. Primary outcomes included PnaP results with respect to utilization appropriateness. Statistical analyses included chi squared testing with p-value < 0.05 considered statistically significant.

**Figure:**
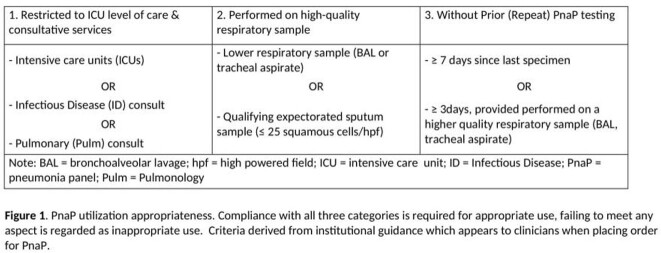

**Results:**

PnaP testing was performed on 574 respiratory samples from 440 patients (Table 1). Overall compliance with institutional guidance was 84% (n=482/574) but varied with respect to level of care (ICU 89% vs non-ICU 72%, p < 0.001) and location (Table 2). Repeated testing was the most common reason for non-compliance, accounting for 59% of inappropriate use. Positivity rates varied significantly respect to guideline compliance and level of care, with distinct pathogen isolation profiles seen among ICU and non-ICU patients. Pathogen isolations was not significantly impacted by respiratory sample type (Table 3).

**Figure:**
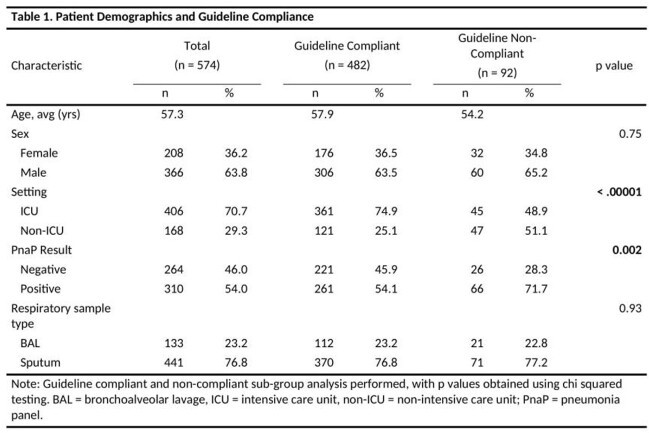

**Figure:**
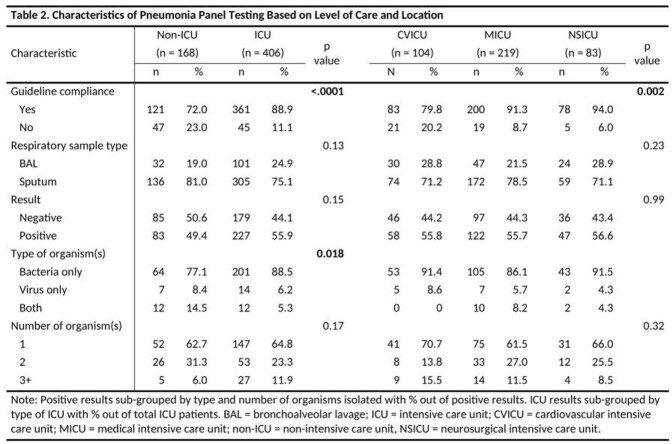

**Conclusion:**

Multiplex panels can isolate significant more pathogens compared to traditional diagnostic testing, with the potential to identify additional variables that factor into pneumonia pathology. PnaP assay results and appropriateness of use appear intertwined, with criteria compliance significantly impacting positivity rates, with distinct pathogens isolated among ICU and non-ICU patient groups. Our study highlights specific target areas for intervention, with the potential to promote clinically appropriate diagnostic testing by adjusting ordering restrictions at our hospital.

**Disclosures:**

**Trevor C. Van Schooneveld, MD**, bioMerieux: Advisor/Consultant|bioMerieux: Grant/Research Support|Insmed: Grant/Research Support|Merck: Grant/Research Support|Thermo-Fischer: Advisor/Consultant.

